# Participatory Training in Monitoring and Evaluation for Maternal and Newborn Health Programmes: Evaluation of a Training Programme Conducted in Africa and South-East Asia

**DOI:** 10.5539/gjhs.v7n2p192

**Published:** 2014-10-28

**Authors:** Jacqueline S. Bell, Debbi Marais

**Affiliations:** 1 Immpact, Division of Applied Health Sciences University of Aberdeen, UK; 2 Division of Applied Health Sciences, University of Aberdeen, UK

**Keywords:** continuing professional development, low and middle income countries, maternal and newborn health, monitoring and evaluation

## Abstract

In the context of slow progress towards Millennium Development Goals for child and maternal health, an innovative participatory training programme in the monitoring and evaluation (M&E) of Maternal and Newborn Health programmes was developed and delivered in six developing countries. The training, for health professionals and programme managers, aimed: (i) to strengthen participants’ skills in M&E to enable more effective targeting of resources, and (ii) to build the capacity of partner institutions hosting the training to run similar courses. This review aims to assess the extent to which these goals were met and elicit views on ways to improve the training.

An online survey of training participants and structured interviews with stakeholders were undertaken. Data from course reports were also incorporated. There was clearly a benefit to participants in terms of improved knowledge and skills. There is also some evidence that this translated into action through M&E implementation and tool development. Evidence of capacity-building at an institutional level was limited.

Lessons for professional development training can be drawn from several aspects of the training programme that were found to facilitate learning, engagement and application. These include structuring courses around participant material, focussing on the development of practical action plans and involving multi-disciplinary teams. The need for strengthening follow-up and embedding it throughout the training was highlighted to overcome the challenges to applying learning in the ‘real world’.

## 1. Introduction

Progress towards Millennium Development Goals (MDGs) 4 and 5 regarding child and maternal health respectively, are significantly off-track and each year millions of women and children continue to die from preventable causes during pregnancy and childbirth (World Health Organization and UNICEF, 2010). The recent Global Strategy for Women’s and Children’s Health identified ‘strengthening countries’ capacity to monitor and evaluate’ as a key principle in ensuring that interventions and investment aimed at improving maternal health actually translate into tangible results and better long-term outcomes (United Nations Secretary General, 2010). Through monitoring and evaluation (M&E), countries can learn which strategies work and what needs to be improved, enabling resources to be better targeted towards saving lives. The evidence from M&E can inform and influence policy development, best practice and the development of health systems for maternal and newborn mortality reduction and the eventual achievement of MDGs 4 and 5.

Globalisation and advances in technology lead to the continual emergence of new evidence and concepts, often not covered at undergraduate or pre-registration level (Duyff, 1999). Continued education is therefore essential to access current scientific information and keeps abreast of the educational, political and social changes affecting the health care environment (Bennett et al., 2000). The literature indicates that it is important that continuing education should promote problem-solving and critical thinking and not just be lecture-dominated, episodic and non-reinforcing. It should include interaction between learners and providers, be based on current and real-life experience and be responsive to learners’ needs (de Villiers, Bresick & Mash, 2003; Sanders, Murphy-Brennan, & McAuliffe, 2003).

Taking all these aspects into consideration, in 2007 a participatory training programme in the M&E of maternal and newborn health (MNH) programmes was developed by Ipact at the University of Aberdeen in collaboration with the Institute of Tropical Medicine in Antwerp and the London School of Hygiene and Tropical Medicine. Ipact is a research and consultancy group within the University of Aberdeen, which works with partners to provide technical assistance and training for improvement in maternal health in low and middle income countries.

The main purpose of the training programme was to strengthen the M&E skills of national and regional programme managers working in MNH, and to build the capacity of academic partner institutions. Underlying the development were two principles – that implementation would be in collaboration with an academic institution in a developing country, and that the opportunity for these institutions to continue implementing the training programme independently would be provided.

Seven 2-week participatory training courses were held, including two Francophone versions in Morocco and Burkina Faso and five Anglophone versions. A total of 156 participants from 34 developing countries underwent training and six academic institutions hosted courses (Table 1). The training programme was supported by the United Nations Population Fund (UNFPA), which paid the fees for fifteen participants on each course.

**Table 1:** Summary of Training Courses 2008-2010

Country	Dates	Host institution	Participant countries
Morocco	Jan. ‘08	INAS, Rabat	Algeria (1), Benin (2), Burkina Faso (5), Burundi (4), Cape Verdi (3), Comores (2), Cote d’Ivoire (4) Guinea (2), Madagascar (2), Morocco (4), Mauritania (1), Senegal (3)
Uganda	Apr. ‘08	Makerere University, Kampala	Afghanistan (1), Indonesia (1), Kenya (2), North Sudan (3), Uganda (6), Zimbabwe (2)
Indonesia	June ‘08	University of Indonesia, Jakarta	Indonesia (6), Philippines (7)
Burkina Faso	Feb. ‘09	GREFSaD, Bobo Dioulasso	Burkina Faso (7), Chad (5), Congo Brazzaville (4), Gabon (5)
Tanzania	Aug. ‘09	The Danish Centre, Arusha	Botswana (3), Kenya (1), Namibia (5), South Africa (2), Tanzania (3), Uganda (6), Zambia (4), Zimbabwe (4)
Bangladesh	Dec. ‘09	BRACU, Dhaka	Bangladesh (12), Cambodia (4), Vietnam (2)
Uganda	Aug. ‘10	Makerere University, Kampala	Eritrea (1), Lesotho (5), Malawi (3), Namibia (4), Rwanda (3), Sierra Leone (3), Uganda (4)

The participatory training programme was designed around supporting participants to develop an M&E programme based on their own MNH work, and country-specific multi-disciplinary teams were encouraged to attend together to facilitate the process and ensure learning across different professional groups. Targeted theoretical lectures were complemented by practical sessions, with a growing focus on participants’ work plans and tools over the two week period. An online forum was set up for participants to hold discussions after completion of the training programme and the expectation was that the plans developed during the training would be implemented.

In December 2010 a review was conducted, commissioned by the funders, UNFPA. The aim of the review was to determine how the participatory training programme had been perceived, any measurable benefits from it and recommendations to inform the design and direction of ongoing training. The objectives of the review were:

(i) to assess the utility of the training to participants;
(ii) to assess the capacity of host institutions to continue training; and
(iii) to elicit stakeholders’ views on ways to improve the training and gain insights for the continuing enhancement of M&E for MNH.

## 2. Method

The review was conducted by researchers in consultation with an academic advisory group. A mixed methods ‘concurrent transformative’ approach was used (Creswell, Plano Clark, Gutmann, & Hanson, 2003) whereby qualitative and quantitative methods are both used equally and in parallel. Ethical approval was granted by the University of Aberdeen’s College of Life Sciences Ethics Review Board. All information provided by respondents remained confidential to the researchers, and individual respondents were not identifiable.

(i) To address the first objective the review followed Kirkpatrick’s model of evaluating training programmes (Kirkpatrick, 1994) and the four levels of learning and skill acquisition were measured, namely reaction, learning, behaviour and results (Box 1).
(ii) The second objective was addressed following a model for institutional capacity strengthening of pathways, based on a review of the literature on capacity enhancement indicators by the World Bank (Mizrahi, 2003). Four institutional characteristics relevant to the task were selected, namely technical capacity; motivation/leadership; organisational capacity and action (Box 1).
(iii) The third objective was addressed by asking all stakeholders for their views.

**Box 1: Aspects included in the evaluation of the training programme**

Utility of training to participants:

Level 1: **Reaction** – how the participants felt about the training

Level 2: **Learning** – increase in knowledge and skills as a result of the training

Level 3: **Behaviour** - extent of applied learning back on the job or implementation

Level 4: **Results** - effect the participant has on his/her own organisation

Institutional Characteristics Reviewed:

**Technical capacity** refers to the level of technical expertise of individuals and of the institution and aspects to be reviewed included approaches to training; technical skills (e.g. appraising data quality, evaluation design); knowledge of MNH and access to training materials. Indicators used to measure the technical capacity were whether facilitators reported a positive effect on their technical expertise and work, whether they reported that materials were of high quality and whether they used the materials in subsequent teaching.

**Motivation/leadership** refers to the extent to which the individuals and the institution wish to replicate the course or develop further courses and aspects to be reviewed included enhanced motivation of facilitators to take part in future courses; enhanced motivation of lead-facilitators to lead future courses and institutional motivation to replicate the course. The indicators used to measure motivation/leadership were whether facilitators reported a positive effect on their professional motivation to do M&E training from Ipact involvement and indicated a desire to take part in similar M&E training in the future or to lead or host a similar course in the future.

**Organisational capacity** refers to the extent to which the organisation commits resources to running a course and aspects to be reviewed included technical resources; internal financial resources; human resources; logistics and buildings. The indicators used to measure organisational capacity were whether facilitators reported adequate internal (human (teaching, technical expertise, admin), physical (infrastructure, materials) and financial) and external (networks/partnerships for participants/facilitators) resources to run future courses as well and the effect on/gain in resources from Ipact contact

**Action** is the final marker of capacity strengthening and aspects to be reviewed included M&E training of MNH programmes being part of the institutional training strategy; replication of the course and other actions taken which build on the experience of running the course. The indicators used to measure action included the facilitator reporting that hosting/leading similar M&E training is part of their strategy, the effect on their strategy from Ipact contact and whether the institutions that re-ran the course took more responsibility.

### 2.1 Data Collection

The review of the participatory training programme included consultation with stakeholders from the seven participatory training courses: these included participants, facilitators, course organisers, support staff as well as representatives of the host academic institutions, the participants’ employers and funders. All reports filed by training course organisers that included summaries of course evaluations from participants were reviewed and information relevant to the objectives extracted. In addition an opportunistic sample of work conducted during the training and retained by the course organisers was reviewed and all substantive postings from the online forum were included.

All 156 course participants were invited via e-mail to complete a questionnaire (as an attached document or online at Survey Monkey) in English or French, depending on the language of the training. This instrument collected information on the participants’ views of the training and whether they perceived it had an effect on their practices (Box 2).

**Box 2: Main themes in course participants’ survey**

- Strengths, weaknesses, opportunities, threats regarding the training
- Skills developed – related to each learning outcome (‘no change’ to ‘fully achieved’)
- Whether the skills developed on the course have made a difference to working practice
- Any other gains from the course that have been useful
- Any specific changes in work as a consequence of the course – examples, if none why not?
- Any effect on employing organisation as a consequence of the course – examples, if none why not?
- Responses to specific questions on logistics, content and delivery of course
- Advice on improving the course

The survey included ten mostly open questions and two sets of questions about each learning objective of the course, whether it had been met and whether the learning had affected the participant’s practice. The responses relating to learning objectives used 5-point Likert scales of 0 to 4 (a ‘not applicable’ option was also available).

The in-country facilitators for each participatory training course were invited to take part in a semi-structured interview either face-to-face or by telephone (Box 3). Representatives from three UNFPA country offices and one regional centre, the two main international facilitators as well as two administrative and business personnel from Ipact were also invited to take part in similar interviews.

**Box 3: Main themes in the course facilitators’ interview**

- Opinion on the course – structure, quality of materials, quality of teaching
- Interaction with international facilitator; adaptation of materials
- Effect of facilitating the course on their own technical capacity
- Any difference to their own practice at work
- Any other benefits from working with the course team
- Whether they would like to be involved in leading/facilitating similar M&E training in the future?
- Whether their institution has adequate resources to run a future course
- Their perspective on the collaboration between Ipact and their institution
- Advice on improving the course – particularly with reference to follow-up

### 2.2 Data Analysis

Quantitative data from the survey were tabulated to indicate frequencies and mean scores calculated for learning outcome achievement and use of skills. Scores were derived by using an ordinal scale of zero for ‘no change’/‘never used’ to four for ‘fully achieved’/‘use often’ for learning outcome achievement and use of skills respectively. Quantitative data from the course reports were extracted as reported. Qualitative data from the open questions in the survey and the interviews were themed according to the two theoretical frameworks (Kirkpatrick and the World Bank).

## 3. Results

Forty-one participants responded to the survey (26% response rate). Interviews were conducted with ten facilitators, two course organisers and two support staff. All five available post-course evaluation summaries and ten electronic communications from participants were examined. Tables 2 and 3 summarise the responses to questions about each learning objective, whether it had been met and whether the learning had affected the participant’s practice.

**Table 2:**
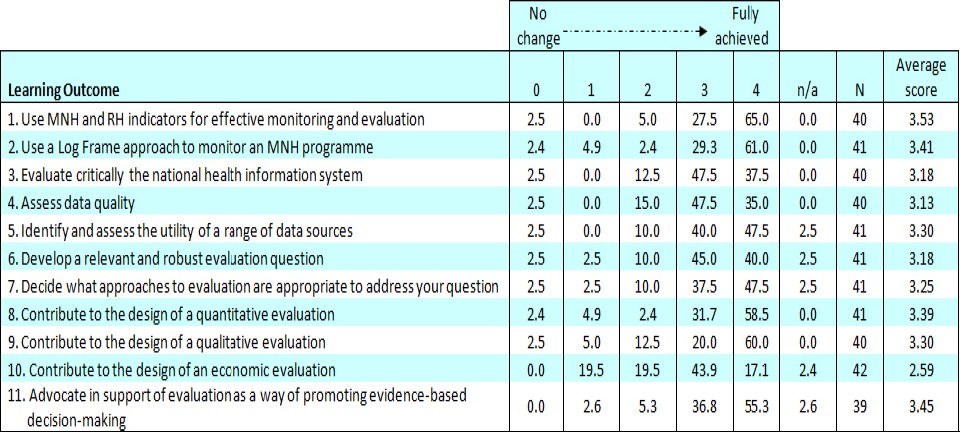
Review Survey - Achievement of learning outcomes, skills development (Percentages)

**Table 3:**
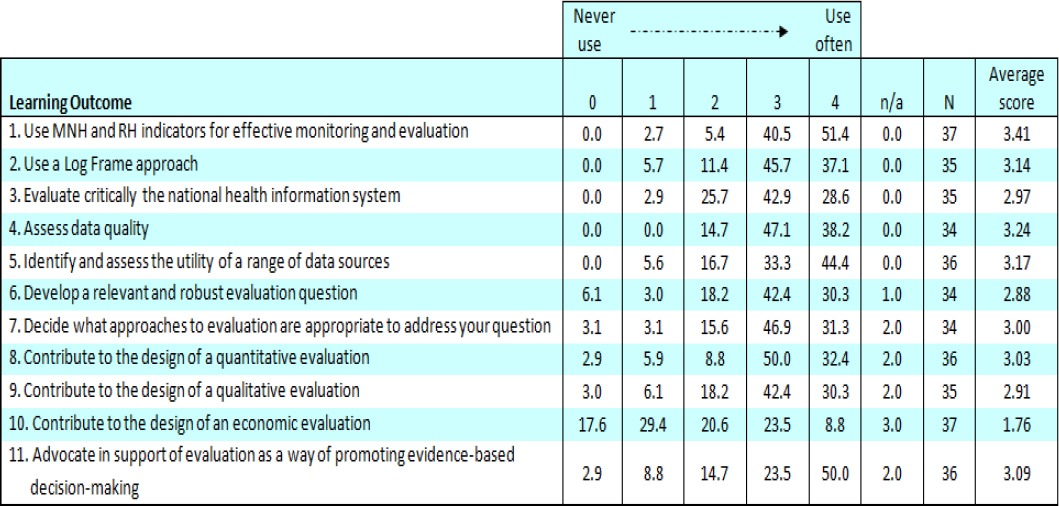
Review Survey - Achievement of learning outcomes, skills development (Percentages)

### 3.1 The Utility of the Training Programme to the Participants

#### 3.1.1 Reaction–How the Participants Felt About the Training

The participants were generally positive in their reaction to the participatory training programme, both immediately after training and in the review process. The strengths most frequently mentioned related to the expertise ‘*lecturers are expert in their field’* (Indonesia) and diversity (multi-disciplinary and local-international composition) of the facilitating teams, using relevant and practical examples within country contexts [*‘… the methodology of training allow practicing according to country status and context’* (Bangladesh)], inter-country exchange or *‘Sharing …lessons learned from other countries’* (Bangladesh) and an interactive and participatory learning environment. Additional advantages reported were the opportunity for networking and building ‘*relationships with other peers for ongoing support’* (Tanzania) and a positive impact on self-esteem ‘*Participants were rightly proud of their work which was of a high standard’* (Uganda).

The weaknesses most frequently mentioned indicated that the programme was extremely full, with insufficient time to deal adequately with all the issues especially in terms of discussions and practical work. The duration of the course was a big discussion-point, with the consensus that it should be shortened. The length of the course was considered to be a disincentive, particularly to government officials. Other suggestions from facilitators included incorporating the course into a Masters programme, training trainers to conduct the courses and offering the training online. Popular suggestions from participants included sending preparatory materials or introducing an introductory online course, along with encouraging countries to bring their own data. Guidelines for running the training and a course manual were both requested. Follow-up after the training was mentioned by most respondents as an area that needed strengthening for the benefit of participants. There were various specific suggestions for implementation including: assigning mentors during the course, monthly emails/Skype from the lead facilitator, follow-up meetings with participants, integrating follow-up into the workplace, and the establishment of a monitoring committee to support countries in implementing their action plans. There was recognition that such innovations would require people assigned to carry them out and associated costs.

The cost of the course and lack of funding opportunities was also often raised as a concern and it was suggested that a reduction in cost would encourage attendance. Other weaknesses mentioned by a few respondents included the challenges posed by the diversity of participants’ language and knowledge level *‘… diversity of participants regarding language barrier and M&E knowledge and skills’* (Bangladesh) and the inexperience and lack of training of some facilitators.

#### 3.1.2 Learning–Increase in Knowledge and Skills as A Result of the Training

The majority of survey respondents felt that the learning objectives for most outcomes were achieved.

The mean scores for 10 of the 11 learning objectives were above 3 (on a scale of 0-4) and reported as ‘fully’ or ‘nearly fully’ met by at least 80% of respondents. For each objective, less than 3% reported no change in their skills, indicating that the objectives were largely achieved.

The objective ‘Use MNH and RH indicators for effective monitoring and evaluation’ was most successful, with 65% of respondents reporting that this was fully achieved. The least successful objective was ‘Contribute to the design of an economic evaluation’ with only 17% of respondents reporting that this was fully achieved and a lower mean score of 2.59. The economic analysis session was also mentioned as the weakest session in some post-course reports, but it was noted that although ‘*the economic evaluation and qualitative approach to evaluation sessions were difficult and unnecessarily detailed*, [they were felt to be] *beyond the level of knowledge that was needed for their work’* (Burkina Faso post-course report).

#### 3.1.3 Behaviour-Extent of Applied Learning Back on the Job–Implementation

Most survey respondents reported that their learning on the course had made a difference to what they do at work (Table 3).

Seven of the 11 objectives had a mean score of more than 3 and another three had mean scores just below 3, indicating that they used what they had learnt fairly often. Again, the objective ‘Use MNH and RH indicators for effective monitoring and evaluation’ was most commonly associated with changed practice, with 51% of respondents reporting that their skills were ‘used often’. As before, the least successful objective was ‘Contribute to the design of an economic evaluation’ with only 9% of respondents reporting that this was used often in their work and the lowest mean score at 1.76. For the economic objective, 18% reported they had never used their skills, whereas this percentage was less than 7% for all other objectives.

When asked whether they had made any specific changes in their work as a consequence of attending the course, responses were substantive. Reported changes included development of health information system (HIS) indicators, development of new tools for data collection, improvement of data quality and analysis of data as indicated by these selected quotes from respondents:

- *From this training I have developed an Excel tool where districts directly integrate the data. That has resulted in the availability of data at the regional level* (Morocco)
- *Yes I now actively participating in a joint project with UNICEF to develop MCH web based data base to collect MCH indicators even if we are just starting* (Uganda 2010)
- *I redesigned indicators to monitor the project “Improving access and quality of reproductive health services in Oyam district in Uganda.” I was able to convince management drop some of the indicators that were either duplicated or were no more useful at different stages of the project* (Uganda 2008)
- *Yes - addressing equity issues in MNCH programme by using disaggregated data by sex, urban/and rural and wealth quintiles* (Tanzania)
- M & E has been emphasized in the different programs and our division is in charge of M & E in one of the global fund projects (Indonesia)

The wider use of logical frameworks and M&E reference documents were also mentioned often. Furthermore, initiatives such as ‘*We hold quarterly meetings to review and validate data for maternal health’* (Morocco) and being recognised as experts in the field were noted.

Improved confidence was reported immediately after the courses ‘*After training, I do have more confidence‘* (Bangladesh)and seems to have been sustained as respondents also indicated in the review that they were more confident. In the survey, respondents reported that the training resulted in them reflecting on their own and others’ work more critically.

Some respondents though indicated that had not had the opportunity to implement what they had learnt but that the course improved their understanding. Barriers to implementing changes were reported as lack of opportunity or time, lack of follow-up, lack of mechanisms for monitoring implementation and the need for support in applying their knowledge after the course.

#### 3.1.4 Results-Effect the Participant Has on His/Her Own Organisation

Beyond the individual behaviour changes already outlined, some additional information was elicited on organisational change. Some respondents reported changes including the finalisation of country strategies and the development of new HIS, including revision of indicators and improvement of data quality as indicated by the following selected quotes:

- *Yes, we have revised our indicators in maternal and child health that we integrated into the national health information system* (Morocco)
- *For the first time, our internal audit of country office programming acknowledged that our M & E system is now strong. A HIS strategy was developed and with effect from 2009, a new system called the District HIS was adopted for use. We anticipate to see an improvement in the quality of data* (Uganda, 2008)
- *This helped in the finalisation of the Sexual and Reproductive Health Strategy which was pending* (Uganda, 2010)

Some respondents reported though that there had been no effect within the organisation or that they were not at a level to initiate change. Some also felt that it was too soon for change to have occurred e.g. following the Uganda 2010 training, just a few months before the review. Barriers impeding change were reported from the course facilitators’ point of view as well as the participants. The restrictive environment on return to their country was seen as a major threat in terms of lack of motivation to implement training, staff mobility and the absence of mechanisms for monitoring the implementation of training. Lack of country ownership was also mentioned by a few respondents.

### 3.2 The Capacity of Host Institutions to Continue Training

#### 3.2.1 Technical Capacity

Most facilitators reported a positive effect on their technical expertise, mainly in terms of knowledge acquired during the course (from content or other facilitators) and increased experience/confidence in facilitating courses. Some facilitators were able to give concrete examples of how their knowledge had been applied subsequently in contributions to projects. Most facilitators reported that materials were of good quality, although none reported using the materials in subsequent teaching. Indirect effects, such as experience of working within a multi-disciplinary group and career development, were also reported.

#### 3.2.2 Motivation/Leadership

All facilitators responded positively to questions on their own motivation to take part in future M&E training programmes, indicating that they found the courses interesting and productive. The national lead-facilitators all expressed a desire to lead similar courses in the future; the importance of the subject area, benefit to participants, professional development and good interactions with participants were the main reasons given. Some did indicate though that the course management load was heavy especially in terms of administrative tasks associated with getting participants to the course, and several indicated they would like more responsibility and involvement in decision-making. Barriers to organising future courses were reported as: having to find funding, a lack of follow-up from Ipact and absence of institutional ‘buy-in’ as there had been no requests for further courses. Several facilitators mentioned that hosting the course had added to the visibility and reputation of the host institution.

#### 3.2.3 Organisational Capacity

In general facilitators reported adequate resources to run future courses in terms of human resources (teaching, technical expertise, administration), physical resources (infrastructure, materials) and networks, but many expressed concern over especially financial, but also time constraints. Some also indicated that they would like to continue to collaborate with Ipact in terms of course delivery and technical expertise, but would not rely on Ipact solely and aimed to be more independent. It was mentioned that this could also improve the credibility of the training and improve networks already established.

#### 3.2.4 Action

Only one partner institution has replicated the course, but the balance of responsibility between Ipact and Makerere University was similar for both courses. However, several of the partner institutions in both African and Asian regions have seriously considered, or are considering, re-running the training. It also seems that other courses may have benefited from the Ipact M&E training (HIV/AIDS and MSc programmes organised by participants). In addition, two future courses are under discussion in Ethiopia and Rwanda.

## 4. Discussion

The two models (Kirkpatrick and World Bank) used to evaluate the training programme were able to assess not only the utility of the training to participants, but also the institutional capacity strengthening, deemed important in the literature on evaluating continued education in health professions (Grace, 2001; Webb, & Sergison, 2003; Middleton et al., 2014). We believe that this dual approach to capacity building in a training programme is important.

It is clear that all four levels of learning and skill acquisition were achieved by participants in the training programme, albeit to varying degrees. Participants were very positive about the training (Reaction). Positive aspects identified were the expertise and multi-disciplinary nature of the facilitating teams, use of relevant and local examples, inter-country exchange/networking opportunities and the participatory learning environment. This sharing of experiences within a diverse inter-professional group having a positive impact has been found to result in positive reactions to other continuing education training within the health care context (Thistlethwaite, 2012; Hammich, Freeth, Koppel, Reeves & Barr, 2007; Webb, & Sergison, 2003; Hook, & Lawson-Porter, 2003). Areas of improvement identified were related to time management and costs especially, and once again such a response is not unique to this training programme (de Villiers, Bresick & Mash, 2003). Almost all the learning objectives were successfully achieved (Learning) with only one being less successful. This economic evaluation was scored lowest both for ‘achieving the learning objective’ and for ‘implementing learning in the workplace’ but it was also reported as the objective of least use in the participants’ work environments.

As noted by others, (Cheng & Hampson, 2008; Grace, 2001) the first two levels are more easily achieved than the next level of application/implementation of the learning in the workplace and levels three and four are not often used because organisations find it much simpler just to focus on the first two levels. The lack of evaluations at this level mean that these levels of evaluation remain poorly understood (Bramley & Kitson, 1994). Most of what the participants had learnt though had been used fairly often (Behaviour) and various changes were reported to have been made in their work because of attending the course. The changes made mostly involved the development of new tools and the improvement of data quality and analysis; participants also gained the advantage of being recognised by their colleagues as experts in the field. The lack of application/implementation though does not seem to be related to the training as such, but rather as a result of participants not having had the opportunity to apply/implement the knowledge. Achievement of the final level, regarding the effect on the participants’ organisations (Results), was the least successful but there were some reported changes in country strategies and systems. This lack of success was again attributed to other factors rather than the training itself. Many indicated that they were not able to initiate changes at their level of seniority, that it was too soon for change to have occurred, competing demands, lack of ownership and accountability and a restrictive environment were also noted as barriers impeding change. This highlights the need to ensure that the most relevant staff are selected for these types of training programmes, so that they are at an appropriate level to be able to initiate change as well as finding ways of ensuring that the employer provides an enabling environment for the employee to be able to implement what they have learnt. Aspects related to the training that impeded change were reported as lack of follow-up, monitoring and support after the course. These are aspects often suggested by course attendees but it is also well-documented that mentorship and support are often not taken up due to time constraints and work-load once participants have returned to the workplace (Hook & Lawson-Porter, 2003). Course manuals, distance learning or a formalised post-graduate programme were also suggested.

The empowerment of participants, with self-reported improvement in self-esteem, self-confidence and reflection seems to be a recurring theme which perhaps does not fit in directly with Kirkpatrick’s four levels. It does though seem to be an essential part of the process and is reported in other evaluations (Sanders, Murphy-Brennan & McAuliffe, 2003; Leigh, Rutherford, Wild, Cappleman, & Hynes, 2013).

The responses from facilitators showed some institutional capacity strengthening in terms of technical capacity and motivation, but there was no evidence they had implemented actions. Most facilitators reported a positive effect on their technical expertise, mainly in terms of knowledge acquired from the course content being applied in contributions to projects, but also gaining confidence in facilitating and working within a multi-disciplinary team. Course administration and institutional buy-in were noted as challenges, and it seems that adequate resources to run future courses are available, but there are funding and time constraints.

Importantly, facilitators indicated that they would like to still collaborate with Ipact in terms of course delivery and technical expertise as this would improve the credibility of the course and improve networks already established, but would not rely on Ipact solely for future courses. Only one partner institution has replicated the course but there was no evidence of an increase in the amount of responsibility they took for the M&E training. Several of the partner institutions have considered, or are considering, re-running the course. Contradicting the lack of institutional buy-in, facilitators mentioned that hosting the course had definitely added to the visibility and reputation of the host institution.

The limitations of the review include its reliance on self-reporting and the low response rate from participants. However these findings, particularly the responses to the qualitative component, help with understanding the processes of the training programme, what was beneficial and what could be improved, and as such have value in the context of the specific programme and may also be instructive for other similar programmes.

## 5. Conclusions

There was clearly a benefit to participants in terms of their learning and behaviour from the participatory M&E training programme provided by Ipact and partners. There is also evidence that this translated into changes at work as well as some M&E implementation, although there were challenges to overcome in the form of lack of capacity and authority to make changes. Evidence of capacity building at an institutional level was limited to the benefit gained by facilitators. One key lesson is that structuring the training programme around material brought by participants seemed to be a successful strategy in terms of engaging participants and facilitating their learning. Designing programmes where the development of practical action plans are the main focus of the training is one way to improve the practicality and applicability of professional development training.

The multi-disciplinary nature of the training was also appreciated by participants and contributed to the development of realistic plans that could be acted on in practice. It was found that consideration of the composition of country-groups was important to ensure that at least some of the participants have the authority to change practice and systems to make use of the knowledge and skills obtained through the training.

Lastly, strengthening follow-up and embedding it throughout the training programme was found to be essential to help overcome the challenges back in the ‘real world’.
